# Induction shouldn't be painful: improving psychiatry local induction for junior doctors across the South West

**DOI:** 10.1192/bjo.2021.99

**Published:** 2021-06-18

**Authors:** Bethany Cole, Harriet Greenstone

**Affiliations:** Avon and Wiltshire Mental Health Partnership NHS Trust

## Abstract

**Aims:**

The GMC recommends that organisations ensure learners have an induction in preparation for each placement. We aimed to ensure that high quality induction was being delivered in psychiatry posts across the whole of the Severn Deanery. This included multiple localities (Bristol, Bath, Swindon, Devizes, Weston-Super-Mare, Gloucester, Cheltenham, Taunton and Yeovil) across three NHS trusts.

**Background:**

Induction plays a vital role in preparing doctors for their new roles. Crucially, some doctors are not only new to the specific role and site, but also new to the specialty (for example, Foundation Doctors and GP Trainees). In Severn, each locality takes responsibility for providing Junior Doctors with a locality-specific induction; these occur four times per year. Previous feedback from trainees in Severn was poor; as demonstrated by informal feedback and the August 2018's GMC survey results, showing some localities ‘required improvement’.

**Method:**

Pre- and post-intervention measurements were ascertained by written questionnaires for Foundation Doctors, GP Trainees and Core Trainees in Psychiatry. Baseline questionnaires were completed in August/September 2019. Five ‘Plan, Do, Study, Act’ Cycles were completed over the following eighteen months. Examples of the changes made included incorporating ‘missed’ topics (such as wellbeing, seclusion reviews and exception reporting) and specific information to on-call responsibilities, reducing replicated information, and touring clinical sites. These changes were coordinated via monthly meetings between Locality Trainee Leads (LTLs).

**Result:**

There was an overall improvement in trainee's satisfaction with induction. Outcomes also included the development of an induction checklist specific to each locality and a ‘gold standard’ list for what local induction should involve. This is hopefully soon to be ratified by the Medical Education department and Severn Deanery.

**Conclusion:**

Having worked on this project for over 18 months, sustainability of change remains a crucial issue. In response to this, we have established several recommendations: the LTL job role needs to be revised to include updating the written induction handbook in each locality and delivering face-to-face induction. Outgoing and incoming LTLs will plan each induction together, at least 4 to 8 weeks before the start date. Support from Medical Education regarding attendees at each induction is to be put in place. Handbooks will be shared across localities, so that the ‘core’ information is consistent. Ongoing feedback will ensure that Junior Doctors continue to receive a high quality and relevant induction.

